# Using Artificial Intelligence to assess the impact of social, physical, and financial health and personality on subjective well-being in a representative, multinational sample of older European and Israeli adults

**DOI:** 10.7189/jogh.15.04179

**Published:** 2025-06-27

**Authors:** Philip J Moore, Germano Vera Cruz, Thomas Maurice, Cynthia A Rohrbeck, Yasser Khazaal, Fallon R Goodman

**Affiliations:** 1 Department of Psychological & Brain Sciences, The George Washington University, Washington DC, USA; 2 Department of Psychology, UR 7273 CRP-CPO, University of Picardie Jules Verne, Amiens, France; 3 Department of Health Economics, University of Poitiers, Poitiers, France; 4 Department of Addiction Medicine, Lausanne University Hospital, Lausanne University, Lausanne, Switzerland

## Abstract

**Background:**

Subjective well-being (SWB) is an important outcome influenced by other aspects of health and personality. However, we know little about the independent effects of multiple health and personality dimensions on SWB in large, representative international samples. Artificial Intelligence (AI) models are particularly well-suited to detect multi-factor patterns in complex topics such as SWB.

**Methods:**

This study involved a representative sample of 37 991 older adults from 17 European countries and Israel. Machine-learning algorithms, general additive modelling, low-degree polynomials (*i.e*. splines), and regression analyses were used to determine the independent effects of the Big 5 personality traits on social, physical and financial health factors, and the impact of all of these on an aggregate measure of SWB.

**Results:**

Loneliness, overall physical health, and making ends meet were the strongest social, physical and financial health predictors of SWB, respectively (absolute value (|t|s) = 29.77–51.53). Neuroticism was a consistent, negative determinant of health (|t|s = 2.82–11.42), but reduced the adverse impact of poor physical health on SWB (|t|s = 4.57–5.98). Extraversion was linked to better social and financial health (|t|s = 2.96–7.74), but also to higher body mass index (Student’s *t* test (t) = 13.52), while openness to experience was positively associated with social and physical health (|t|s = 3.02–7.86), but negatively related to income (*t* = −19.76).

**Conclusions:**

All adverse health factors and neuroticism were linked to lower SWB, while SWB was positively associated with the other health measures and personality traits. Some traits had unexpected effects on health outcomes, and some had moderating effects on the links between these outcomes and SWB, suggesting that the links between personality, health and SWB depend on the types of health considered. Future multivariate modelling is recommended to clarify the mechanisms for these and other observed relationships.

Health and well-being are among the most fundamental, universal, and highly-valued human conditions [1]. Although both are complex, multifaceted states with overlapping components, health and well-being are not identical. Health is associated with a wider range of conditions and responses (*e.g*. physical, social, financial). As defined by Sartorius, health includes the absence of disease or impairment, the resources to cope with the demands of life, and an adaptive balance within individuals, and between them and their environment [2].

Well-being is typically described in more general, holistic terms. Simons and Baldwin defined well-being as ‘a state of positive feelings and meeting full potential in the world’ [3]. This distinction is also reflected in an electronic search conducted in April 2025 which used an Artificial Intelligence (AI) algorithm to generate 100 types of health and well-being, respectively [4]. In each list of 100, 95 types of health contained the word ‘health,’ while only 22 of the well-being types contained the term ‘well-being.’

The summative nature of well-being is particularly evident in subjective well-being (SWB), a broad construct reflecting to how people evaluate and experience activities, conditions, and the overall state of their lives [5]. There are at least two domains of subjective well-being. Evaluative SWB refers to individuals’ perceived life quality, fulfilment or satisfaction, which is measured by both single-item and aggregate assessments [6,7]. Experiential SWB refers to the frequency and intensity of emotional experiences that make a person’s life more pleasant or unpleasant [8]. These positive and negative emotions have long been associated with health [9,10] and survival [11] (Appendix A, section 1, in the **Online Supplementary Document**).

Subjective well-being is also a valuable marker of social progress [12], and a key piece of information for public policy [13]. These benefits are particularly important for older adults (*e.g*. 50 years and older), who represent an increasingly large percentage of the world’s population [14]. Accordingly, SWB has become an increasingly prominent topic of research. A literature search (PsycINFO, APA PsycArticles, MEDLINE, and Academic Search Complete) in April, 2025 identified 830 articles with ‘subjective well-being’ in the title between 1980 and 2002, and over 8100 such articles since 2003.

Just as there are different dimensions of subjective well-being, there are different domains of health, many of which are associated with SWB. For example, physical health factors such as overall health and chronic illness have significant effects on SWB [15,16], as do aspects of cognitive health [17,18]. Behaviours such as diet [19] and physical activity (PA) [20] are also integral to health and strongly related to SWB.

Individual’s social perceptions and experiences (*e.g*. network size and satisfaction) are also valuable aspects of health, with important implications for subjective well-being [21,22]. More distal but no less important, financial factors (*e.g*. income, ability to make ends meet) are similarly impactful components of health [23,24]. Many of these health factors have conceptual overlap, and all of them have the potential to influence each other and SWB. The importance of health and SWB in human life argues strongly for a better understanding of how, when, and for whom these influences operate.

While health and SWB are influenced by many conditions, they are ultimately processed and experienced individually. Thus, very similar circumstances can lead to very different reactions in different people [25]. These differences in people’s responses to their internal and external environments (aka personalities) appear to develop early in life, and they tend to be stable over time [26]. Among the most prominent and well-validated personality measures are the so-called ‘Big 5′ personality traits [27]. These include openness to experience (O) (embracing new ideas and interests), conscientiousness (C) (thorough, disciplined, and considerate of others), extraversion (E) (outgoing, social, and emotionally expressive), agreeableness (A) (trusting, compliant, and cooperative), and neuroticism (N) (tending to view things in negative and/or unstable ways).

Given their early onset and enduring nature, personality traits may also play a role in human health. In fact, openness, conscientiousness, extraversion, and agreeableness are typically associated with better health and well-being, while neuroticism tends to be negatively related to these same outcomes [28–30]. Beyond their direct effects on health and SWB, personality traits may also exert indirect (*i.e*. moderating) effects by influencing the impact of health-related factors on SWB. For example, Potter et al. [31] found that more extraversion weakened the positive relationship between cognitive processing speed and life satisfaction (Appendix A, section 2, in the **Online Supplementary Document**).

Previous SWB research faced many practical and methodological constraints that pose a number of issues regarding their results. First, these studies were necessarily conducted at different times, in different settings, and with different protocols and procedures, making it difficult to identify or control for contextual effects. Second, these studies typically included convenience samples from available academic, community or clinical cohorts, limiting their representativeness, especially to older adults, on whom there is a relative dearth of research [32]. Finally, sample sizes typically ranged in the hundreds, limiting statistical power. Depending on effect sizes and other parameters, the power necessary to assess the unique impact of 50 or more factors may require thousands of participants [33].

To address these issues, Vera Cruz et al. [34] used AI to examine 50 SWB predictors in a stratified random sample of older adults from 17 European countries and Israel. Vera Cruz et al. [34] found that:

1) loneliness was the strongest single SWB predictor;

2) social, physical, and financial health were the strongest categorical predictors;

3) the effects of personality traits on SWB were – in descending order – neuroticism (−), extraversion (+), conscientiousness (+), agreeableness (+), and openness (+).

These results are part of a small but growing literature using AI to examine SWB and its determinants. This includes research finding that all ‘Big 5′ personality traits predicted life satisfaction [35] with the same pattern reported by Vera Cruz et al. [34]. Kaiser et al. [36] found that life satisfaction decreased until age 50, after which it increased with age. Prati [37] found that financial sufficiency, overall physical health, and country of residence were the top three overall predictors of quality of life, life satisfaction, and happiness.

Previous AI research on SWB also had a number of limitations. These included a lack of positive experiential SWB among aggregate SWB measures, which limited their content validity. These studies also have yet to adjust for increased Type I error associated with the many analyses made possible by AI and large databases. Nor have these studies examined the impact of personality on health, or how they combine to determine subjective well-being.

These limitations pose a number of fundamental questions. First, how strongly do personality traits influence different aspects of health? Also, to what extent do these traits enhance – or limit – the impact of these health factors on subjective well-being? The answers to these questions can identify individuals who are more (or less) at risk for poor health or low SWB, and may uncover avenues of intervention for improving SWB.

Examining direct and indirect effects of personality and health on SWB may also help explain how these factors determine subjective well-being. For example, personality and health may interact to significantly influence SWB, even if neither exerts a direct effect on its own. Similarly, when these factors do impact SWB directly, their moderating effect may influence SWB in the opposite direction. By showing how personality, health, and other factors combine to influence each other and SWB, this research can also improve interventions designed to promote these important outcomes.

Using the same two databases as Vera Cruz et al. [34], this study employed AI, generalised additive modelling and multiple regression to assess SWB across countries, categories, and individual predictors. It also examined the impact of personality on social, physical and financial health, and the direct and indirect effects of these factors on subjective well-being. The aggregate SWB measure incorporated both positive and negative experiential subjective well-being, and the results were adjusted to reduce Type I error. These methods were combined to address the following questions:

1) How is SWB distributed across Europe, Israel and the world?

2) How do social, physical, and financial health factors influence SWB?

3) How does personality influence social, physical, and financial health, and SWB?

4) How do social, physical, and financial health combine with personality to determine SWB?

## METHODS

### The study data

The data for this study come from the Survey on Health Aging and Retirement in Europe (SHARE), a large-scale, ongoing programme developed and administered by a team of researchers, clinicians and statisticians in 26 European countries and Israel [38]. The SHARE survey has been conducted since 2004, and includes longitudinal interviews with representative samples of European and Israeli adults aged 50 and older [39,40]. Participants are typically interviewed every two years until their death, at which point an end-of-life survey is offered to their relatives to learn about the end of participants’ lives.

To maximise consistency for each wave, a common questionnaire and protocol are developed by the SHARE European Infrastructure Consortium (ERIC), coordinated in each country by the SHARE Central Coordination Team (CCT), and administered by each SHARE Country Team (CT). The questionnaires are translated into each language using a web-based translation tool, and edited by native speakers to ensure their relevance and accuracy (https://share-eric.eu/). SHARE teams conduct face-to-face interviews using laptops with computer-assisted personal interviewing (CAPI) software [41]. While most of the data are measured directly, data from country-specific systems (*e.g*. education, occupation, income) are transformed using international scales to ensure comparability.

Once collected from participating countries, the data are aggregated and processed for quality control (*e.g*. identifying and eliminating errors), harmonisation (*e.g*. confirming comparability across countries), and missing data (using the hot-deck imputation method). The latest SHARE data available at the onset of this research was Wave 7, which was a ‘special edition’ that included childhood experiences. Thus, this study included Waves 6 and 7 (2014 and 2016) to provide both past and current information. The top and bottom 1% of values were trimmed from the country-specific distribution of each variable to exclude outliers, which were then imputed using the hot-deck method.

The study includes data from 17 European Union (EU) countries (Austria, Belgium, Croatia, Czech Republic, Denmark, Estonia, France, Germany, Greece, Italy, Luxembourg, Poland, Portugal, Slovenia, Spain, Sweden, and Switzerland) and Israel, and a total of 37 991 participants. Participants – 21 412 (56.4%) of whom were female – ranged in ages from 50 to 102 years, with a mean age of 66.1, a median age of 65, and a standard deviation of 9.7 years.

### Subjective well-being

The current measure of subjective well-being combined four indices of SWB. The first was a single-item assessment of life satisfaction, which asked participants, ‘all things considered, how satisfied are you with your life as a whole?” on a scale from 0 (completely dissatisfied) to 10 (completely satisfied). This single-item index has been used extensively to assess life satisfaction [42], and its construct validity has been affirmed by its association with the Satisfaction with Life Scale (SWLS) [43].

The second component – quality of life (QoL) – was assessed using the 12-item Quality of Life Scale (CASP-12), a shortened version of the CASP-19 Quality-of-Life Scale [44]. Participants indicated how often – from 0 (never) to 3 (often) – they had had 12 experiences (*e.g*. ‘I look forward to each day’). Responses to negative items (*e.g*. ‘I feel left out of things’) were reverse-coded, and possible scores ranged from of 0 to 36, with higher scores indicating higher QoL. This measure had high internal reliability (Cronbach’s = 0.86) and a strong association with SWB, and is used widely in research among older adults [5,44].

The third SWB component was depression, as assessed by the Euro-D Depression Scale (EDDS), a shortened version of the 14-item EDDS [45]. Participants indicated whether or not they had experienced each of 12 depressive symptoms during the previous month, resulting in possible scores of 0 to 12, with higher scores indicating more depression. EDDS scores were reverse-coded before being combined with other SWB components. Depression has a profound impact on SWB [46], and EDDS has high internal reliability (Cronbachs >0.80) [47].

While previous SHARE studies have used the three components above [48–50], happiness was added to the current SWB measure to increase its content validity (by including a positive evaluative SWB). Happiness was assessed by whether or not participants had experienced distinct periods of happiness. This unrestricted timeframe was used to maximise capture of happiness (by not limiting it to a particular time period), while minimising participant burden with a single item. Similar dichotomous happiness measures have been significantly associated with subjective well-being [51].

A principal component factor analysis was conducted on the SWB components using SPSS 29.00. This unrotated analysis yielded the following factorial structures: component 1 (eigenvalue = 2.05); component 2 (eigenvalue = 0.97); component 3 (eigenvalue = 0.57); and component 4 (eigenvalue = 0.39). Component 1 – used for the current SWB measure – explained 51.3% of the variance, and correlated significantly with life satisfaction (correlation coefficient (r) = 0.85), QoL (r = 0.80), depression (r = −0.78), and happiness (r = 0.23), respectively. Assumptions of adequacy (Kaiser-Meyer-Olkin (KMO) = 0.678) and sphericity (Bartlett’s test degree of freedom (6) = 27 898.77, *P* < 0.001) were also met. Items for this measure were centred, standardised, and averaged to create a composite index of subjective well-being (mean (x̄) = 0, median (MD) = 0.08, standard deviation (SD) = 0.97, minimal (min) = −5.01, maximal (max) = 1.74).

### Happiness and SWB

Noting its relatively weak loading on SWB, we analysed SWB with and without happiness. Adding happiness resulted in very similar SWB rankings across countries (r = 0.96, *P* < 0.001). The only changes were between Belgium (from 7th to 8th) and Slovenia (from 8th to 7th), and between France (from 10th to 12th) and Israel (from 12th to 10th), neither of which were statistically significant. The only *P*-value changes of 0.01 or greater among SWB predictors were for social contact frequency (from 0.01 to 0.02), social network size (from 0.03 to 0.04), and body mass index (BMI) (from 0.06 to 0.03). Happiness weakened the links between agreeableness and SWB (*P* < 0.001 to *P* < 0.01) and extraversion and social contact frequency (from *P* < 0.05 to *P* < 0.10). However, it increased the moderating effects on SWB of extraversion and loneliness (*P* < 0.05 to *P* < 0.10) and conscientiousness and loneliness (*P* < 0.01 to *P* < 0.001). Given the small number and sizes of these differences, the four-part SWB was used in this research.

### Health factors

Because social, physical and financial health were the strongest categorical predictors of SWB in Vera Cruz et al. [34], this research focused on factors from these three categories. Although these factors all reflected their respective categories, many factors were relevant to more than one category. Thus, categorical designations were made in accordance with the primary themes of the research. For example, loneliness was included in social health, BMI in physical health, and inability to afford food was viewed as a financial health factor.

### Social health

Six social-health factors were among the top 50 SWB predictors, including loneliness, which was self-assessed on a scale from 0 (not at all) to 6 (very lonely). Participants’ social network satisfaction and social activity satisfaction were measured on a scale from 0 (completely dissatisfied) to 10 (completely satisfied), while social contact frequency was either 0 (never), 1 (once a year), 2 (once a month), 3 (twice a month), 4 (once a week), 5 (twice a week), or 6 (daily). Social network size reflected the number of people participants reported in their social network, and their social network proximity was reported as either 1 (>100 km (km)), 2 (25–100 km, inclusive), 3 (1–25 km), or 4 (<1 km).

### Physical health

The five physical-health factors among the 50 SWB predictors included general health, indicated by participants as either 1 (poor), 2 (fair), 3 (somewhat good), 4 (good), or 5 (excellent). Participants reported whether they suffered from a chronic disease (1) or not (0), as well as their number of chronic diseases. Participants’ limited physical activity due to their health was rated as 1 (no limitation), 2 (some limitation), or 3 (severe limitation). Participants’ body mass index (BMI) was calculated by dividing their weight in kilograms by their height in meters squared (kilogrammes per square metre (kg/m^2^)).

### Financial health

The six financial-health factors included annual participant income. Due to many missing values, imputation was conducted for this variable by the SHARE team using the hot-deck method. Income was adjusted for the relative prosperity of each country and the exchange rate between each currency and the euro in 2015. Employment status was recorded as 1 (retired, unemployed, permanently disabled), 2 (homemaker), or 3 (employed). Ability to make ends meet was assessed from 1 (difficult) to 4 (easy), while the final three financial health factors were whether participants were ever unable to afford either heat, food, or health care, each of which was assessed as either 1 (yes) or 0 (no).

### Personality traits

The five personality traits in this research were assessed using at least two statements (one positive and one negative) regarding the trait, using the following five-point scale:

1) ‘strongly disagree,’

2) ‘disagree a little,’

3) ‘neither agree nor disagree,’

4) ‘agree a little,’ or

5) ‘strongly agree.’

Responses to negative-oriented items were reverse-scored before being combined with other responses.

### Neuroticism

The two statements used to assess neuroticism were:

1) ‘I see myself as someone who gets nervous easily,’ and

2) ‘I see myself as someone who is relaxed and handles stress well.’

### Extraversion

The two statements used to assess extraversion were:

1) ‘I see myself as someone who is outgoing and sociable,’ and

2) ‘I see myself as someone who is reserved.’

### Conscientiousness

Conscientiousness was assessed using:

1) ‘I see myself as someone who does a thorough job,’ and

2) ‘I see myself as someone who tends to be lazy.’

### Agreeableness

Three items were used to assess agreeableness:

1) ‘I see myself as someone who is generally trusting,’

2) ‘I see myself as someone who is considerate and kind to almost everyone,’ and

3) ‘I see myself as someone who tends to find fault with others.’

### Openness to experience

The two statements used to assess openness to experience were:

1) ‘I see myself as someone who has an active imagination,’ and

2) ‘I see myself as someone who has few artistic interests.’

### Data analyses

#### Rank-ordered SWB predictors

To rank-order the 50 strongest SWB predictors, we used the following machine-learning algorithms: The (Variable Selection Using Random Forests (VSURF) algorithm [52] for variable selection, and the Random Forest algorithm [53] to determine the rank-ordered predictors (Appendix, section 4 in the **Online Supplementary Documen**t). This machine-learning approach has a number of advantages over traditional statistical procedures. Artificial Inteligence algorithms continuously learn about patterns in the data, so they can model relationships without having their form specified a priori. Using distributed computing frameworks, AI is also better able to scale to large data sets.

#### Effect sizes and significance

To determine effect sizes and significance of the SWB predictors, we applied Generalized Additive Modeling (GAM) to the continuous and ordinal data [54,55], and Analysis of Variance (ANOVA) to nominal predictors. To adjust for potential Type I error, the False Discovery Rate (FDR) was applied to the inferential analyses. The FDR involves ranking observed *P* values from largest to smallest, then multiplying each *P*-value by N/N-k, where N equals the number of comparisons, and k the respective ranking – from 1 to N – of each successive *P*-value. If the adjusted *P*-value is less than the specified alpha level (0.05 in this research), the difference is determined to be significant, and the null hypothesis is rejected [56].

### Sensitivity analysis

To assess the impact of machine learning *vs*. standard regression analyses on SWB predictors, a sensitivity analysis was conducted by calculating a Partial Dependence Plot (PDP) score for these two approaches. This PDP score low-to-moderate (0.249), indicating that the analytic method had a noticeable, but not particularly large effect on the outcome.

#### Personality and health

To assess the impact of personality on the social, physical, and financial health factors, 17 multiple-regression analyses were conducted in which each individual health factor was regressed on all five personality traits. Each analysis controlled for the other 16 health factors, as well as for participant age, sex, education, and country of residence.

#### Personality, health and SWB

The combined (*i.e*. moderating) effects of personality and health on SWB were assessed by determining the direction and significance of the interactions for each of the personality measure and health factors, after adding these interaction terms to each of the regression equations indicated above.

## RESULTS

### SWB across countries

#### SWB in Europe

**Figure 1:**
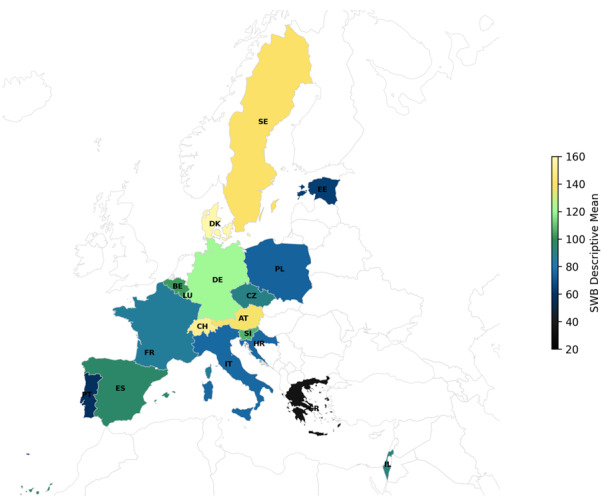
Subjective well-being across Europe. Heatmap showing subjective well-being (SWB) tiers across European countries based on 2016 SHARE survey data. The lowest SWB tier is in black, and the highest is in yellow.

National sample sizes range from 340 (Poland) to 4579 (Estonia). Denmark had the highest SWB (0.580), and Greece the lowest (−0.648), and the overall eta-squared for the 18 countries was partial eta squared (η^2^_p_) = 0.093, *P* < 0.001. Post-hoc Bonferroni tests indicated eight tiers of countries – *a* through *h* – that differed significantly in terms of SWB (**Figure 1**). These included, in order, Denmark/Switzerland, Sweden/Austria, Luxembourg/Germany, Slovenia/Belgium/Spain, Spain/Israel/Czech Republic/France, Italy/Croatia/Poland, Poland/Estonia/Portugal, and Greece. Northern European SWB (x̄ = 0.286) was significantly higher than in Southern Europe (x̄ = −0.319) (Student’s *t* test, degrees of freedom (8) = 4.32, *P* < 0.01)), while Eastern European SWB (x̄ = −0.023) did not differ significantly from the other two European regions (practical significance >0.30) (Table S1 in Appendix B in the **Online Supplementary Document**).

Subjective well-being was unrelated to sample sizes across the 17 European countries (*P* > 0.88) and the three European regions (*P* > 0.50). Although countries’ SWB scores were uncorrelated with their life expectancies (*P* > 0.29), life expectancies were significantly associated with the eight SWB tiers (r, degrees of freedom (8) = 0.56, *P* < 0.05). Life expectancies were also significantly higher in Northern than Southern Europe (t(8) = 4.37, *P* < 0.01), with no significant differences between these groups and Eastern Europe (practical significance >0.34).

#### Well-being around the world

**Figure 2:**
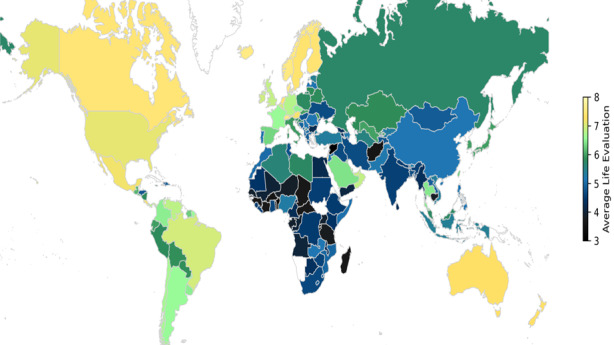
Well-being around the world. Heatmap showing well-being tiers around the world based on the 2016 World Happiness Index (WHI). The lowest WHI tier is in black, and the highest is in yellow.

Although SWB scores were specific to Europe and Israel, they were highly correlated with the 2016 World Happiness Index (WHI) scores [57] for the same countries (r(18) = 0.94, *P* < 0.0001). Thus, as a proxy for SWB, we compared WHI scores across 15 regions around the world (**Figure 2**). The WHI is comprised of a baseline (‘dystopia’) score and seven other components, including per capita GDP, social support, healthy life expectancy, freedom to make life choices, generosity, perceived corruption, and residual variance (Section 5 in Appendix A and Table S2 in Appendix B in the **Online Supplementary Document**).

Countries in these world regions ranged from 1 (Russia) to 36 (sub-Saharan Africa). World Happiness Index scores ranged from a high of 7.285 (Oceania) to a low of 4.261 (sub-Saharan Africa), with an overall eta-squared of 22.19, *P* < 0.001. Post-hoc Bonferroni tests revealed six regional tiers – *a* through *f* – that differed significantly in WHI. These tiers included, in order, Oceania/North America, North America/North Europe, South America, East Asia/Central America/Middle East/Southeast Asia/South Europe/East Europe/Central Asia, North Africa/South Asia, and South Asia/Sub-Saharan Africa. World Happiness Index scores were unrelated to the number of countries within regions (practical significance > 0.27), but WHI scores did predict life expectancies across regions (r(15) = 0.78, *P* < 0.001), and even more highly across regional tiers (r(8) = 0.86, *P* < 0.0001).

### SWB across categories

Nine of the 15 original SWB predictor categories had two or more factors among the top 50 individual SWB predictors. Categorical rankings reflected the average rank – from 1 to 50 – of the individual factors within each category, with lower means reflecting higher rankings (Table S3 in Appendix B in the **Online Supplementary Document**). All six social health factors were among the top 50 SWB predictors, and social health had the highest categorical ranking (the percent increase in Mean Squared Error (%IncMSE) = 9.17). Physical and financial health had rankings of 14.80 and 18.33, respectively. All five personality traits were among the top SWB predictors, with an average ranking of 22.00, a ranking shared by three of the five demographic factors (age, sex, education). The last four categories (health behaviours, living environment, childhood experiences, health care) all had mean rankings of 30 or above.

Individual Mann-Whitney comparisons revealed two tiers of categorial SWB predictors in terms of %IncMSE ranking, with the last three being significantly weaker predictors of SWB than the others, except health behaviours. To maximise their relevance to subjective well-being, only the 25 SWB predictors from the top five categories were included in subsequent analyses.

### SWB predictors

#### Descriptive results

Overall, 23 of the 25 SWB predictors included 37 991 observations. Three of the top 10 SWB predictors – including the top two – were aspects of social health, while three were physical health, two were financial health, and one was a personality trait (Table S4 in Appendix B in the **Online Supplementary Document**). Loneliness was the top social health factor (x̄ = 0.88 on a 0–6 scale), followed by social activity satisfaction (x̄ = 8.00 out of 10) and social network satisfaction (8.96 out of 10). Participants reported an average of 2.84 (out of 5) for general health and 1.75 chronic diseases, and 46% of participants reported limited activity due to health. Participants also reported being able to make ends meet most of the time (x̄ = 2.89 out of 4), with average and median incomes of 98 570 and 26 505 euros, respectively.

Social contact frequency averaged about three times a month (x̄ = 4.81), social network proximity a little over one km (x̄ = 3.32), and social network size averaged 2.69 contacts. Over half (52.9%) of participants had a chronic illness, and their average BMI was somewhat overweight (x̄ = 27.11). About 5.3% of respondents were not able to afford food at some point. Mean personality ratings were lowest for neuroticism (x̄ = 2.62), followed by openness to experience (x̄ = 3.35), extraversion (x̄ = 3.51), agreeableness (x̄ = 3.70), and conscientiousness (x̄ = 4.11).

#### Predicting SWB

Percent increase in Mean Squared Error (%IncMSE) ranged from a high of 191.34 (loneliness) to a low of 3.72 (cannot afford food). Only loneliness and social activity satisfaction had MSE scores of over 100, while all other SWB predictors had scores of less than 75 (**Table 1**). Thirteen (52%) of these predictors had scores of over 20, eight (32%) of the scores were between 10 and 20, and the remaining four (16%) of scores were below 10.

**Table 1:** Inferential results for predictors in top three SWB predictor categories (social factors, physical health, financial status), demographics, and personality characteristics

**SWB predictors**	**Rank**	**Cat**	**%InMSE**		**+/−**	**Effect (F)**		***P*-value**
Loneliness	1	8	191.34	-			2655.02	<0.001
Social activity satisfaction	2	8	128.62	+			1281.83	<0.001
Self-rated general health	3	9	72.18	+			914.95	<0.001
Making ends meet	4	7	68.71	+			886.26	<0.001
Neuroticism	6	14	59.45	-			545.22	<0.001
Social network satisfaction	7	8	52.87	+			162.75	<0.001
Income	8	7	36.26	+/−			14.61	<0.001
Limited activity due to health	9	9	34.14	-			167.52	<0.001
Number of chronic diseases	10	9	31.02	-			118.41	<0.001
Social contact frequency	12	8	24.52	+			1.32	0.02
Extraversion	13	14	21.57	+			40.97	<0.001
Can’t afford health care	14	7	20.98	-			1923.91	<0.001
Age	15	1	20.12	-			26.01	<0.001
Social network distance	16	8	19.83	-			22.17	<0.001
Social network size	17	8	19.02	+			4.23	<0.04
Employment status	18	7	17.55	+			16.87	<0.001
Can’t afford heat	20	7	15.91	-			1679.43	<0.001
Have a chronic illness	21	9	15.40	-			3151.07	<0.001
Sex	22	1	14.77	F>M			588.60	<0.001
Conscientiousness	24	14	14.07	+			84.14	<0.001
Education	29	1	11.82	+			15.89	<0.001
Agreeableness	33	14	9.44	+			1.95	0.096
Openness to experience	34	14	8.89	+			4.53	<0.05
Body mass index	41	9	6.11	-			4.85	<0.05
Can’t afford food	46	7	3.72	-			126.76	<0.001

N – sample size, M – composite SWB index mean, n/a – not applicable, SD – standard deviation

The vast majority (80%) of the SWB predictors were significant at the *P* < 0.001 level, four (16%) were significant at the *P* < 0.01 level, and one (agreeableness) was only marginally significant (*P* < 0.10). Most effects were in the expected direction, although some were less intuitive. For example, participants’ SWB decreased as they got older (Fisher F-test (F)1.94, 37989) = 25.39, *P* < 0.001), females reported significantly higher SWB than males (F(1.37989) = 336.46, *P* < 0.001), and SWB increased with annual income up to 350 000 euros, and then decreased with incomes of greater than 500 000 euros (F(1.95, 37989) = 13.37, *P* < 0.001).

### Personality and health

#### Social health

As shown in **Table 2**, 14 (47%) of the 30 tests for effects of personality on social health were statistically significant. Loneliness was associated with more neuroticism (*t* = 5.45, *P* < 0.001) and less extraversion (*t* = −5.85, *P* < 0.001). Participants who were more extraverted (*t* = 2.96, *P* < 0.01) and open to experience (*t* = 6.30, *P* < 0.001) were more satisfied with their social activity. Greater social network satisfaction was associated with more extraversion (*t* = 4.05, *P* < 0.001), conscientiousness (*t* = 5.43, *P* < 0.001), and agreeableness (*t* = 4.75, *P* < 0.001). More agreeable participants were in more frequent contact with their social network (*t* = 4.10, *P* < 0.001), and those with greater neuroticism lived further from their network (*t* = -7.90, *P* < 0.001). Social network size was negatively related to neuroticism (*t* = −5.03, *P* < 0.001), and positively associated with conscientiousness (*t* = 6.42, *P* < 0.001), extraversion (*t* = 2.67, *P* < 0.05), agreeableness (*t* = 3.77, *P* < 0.001), and openness to experience (*t* = 7.86, *P* < 0.001).

**Table 2:** Main effects of personality on SWB and social, physical, and financial health*

		Impact of personality (t-scores)				
**Category and predictor names**	**N**	**Neuro-ticism**	**Extra-version**	**Conscien-ciousness**	**Agreeabl-eness**	**Openness**
Subjective well-being	37 991	−31.64†	10.87†	12.79†	2.94‡	3.15‡
		ns	ns	ns	ns	ns
Social Health						
*Loneliness*	37 991	5.45†	−5.85†	ns	ns	ns
*Activity satisfaction*	37 991	ns	2.96†	ns	ns	6.30†
*Network satisfaction*	37 991	ns	4.05†	5.43†	4.75†	ns
*Contact frequency*	37 991	ns	ns	ns	4.10†	ns
*Network proximity*	19 359	−7.90†	ns	ns	ns	ns
*Network size*	19 359	−5.03†	6.42†	2.67§	3.77†	7.86†
		ns	ns	ns	ns	ns
Physical health						
*Overall health*	37 991	ns	ns	4.82†	ns	ns
*Activity limitations*	37 991	ns	ns	ns	ns	ns
*Chronic diseases*	37 991	9.55†	ns	−4.84†	ns	ns
*Chronic illness (yes/no)*	37 991	ns	ns	ns	ns	ns
*BMI*	37 991	8.16†	13.52†	−17.89†	−5.28†	−3.02‡
		ns	ns	ns	ns	ns
Financial Health						
*Making ends meet*	37 991	−3.88†	7.74†	ns	ns	ns
*Income*	37 991	−11.42†	7.69†	ns	19.33†	−19.76†
*Employment*	37 991	ns	5.03†	2.32§	2.54§	ns
*Cannot afford care*	37 991	ns	ns	ns	ns	ns
*Cannot afford heat*	37 991	2.82†	−3.97†	ns	ns	ns
*Cannot afford food*	37 991	ns	ns	−3.93†	ns	4.02†

ns – non-significant

*Each *P*-value reflects the False Discovery Rate (FDR) adjustment for multiple comparisons.

†*P* < 0.001.

‡*P* < 0.01.

§*P* < 0.05.

#### Physical health

Personality traits had a significant impact on 8 (27%) of the physical health factors. More neurotic participants had more chronic diseases (*t* = -5.85, *P* < 0.001), and those who were more conscientious had fewer chronic diseases (*t* = −4.84, practical significance <0.001) and had better overall health (*t* = 4.82, practical significance <0.001). Neuroticism and extraversion were positively associated with BMI (*t* = 8.16 and *t* = 13.52, practical significance <0.001, respectively), and BMI was lower among those who were more conscientious (*t* = −17.89, *P* < 0.001), agreeable (*t* = −5.28, *P* < 0.001), and open to experience (*t* = −3.02, *P* < 0.001).

#### Financial health

Thirteen (43%) of the links between the personality and financial health factors were statistically significant. Inability to afford health care was unrelated to any personality factor (practical significance >0.37). Participants with higher neuroticism were more likely to be unable to afford heat (*t* = 2.82, *P* < 0.001), and less likely to make financial ends meet (*t* = −3.88, *P* < 0.001). The opposite was true for more extraverted participants, which were less likely to be unable to afford heat (*t* = −3.97, *P* < 0.001), and more likely to make financial ends meet (*t* = 7.74, *P* < 0.001). Inability to afford food was negatively related to conscientiousness (*t* = −3.93, *P* < 0.001), and positively correlated with openness to experience (*t* = 4.02, *P* < 0.001). Participants who were more extraverted (*t* = 5.03, *P* < 0.001), conscientious (*t* = 2.32, *P* < 0.05), and agreeable (*t* = 2.54, *P* < 0.05) were more likely to be employed. Income was lower among more participants with more neuroticism (*t* = −11.42, *P* < 0.001) and openness (*t* = −19.76, *P* < 0.001), and higher for those more extraverted (*t* = 7.69, *P* < 0.001) and agreeable (*t* = 19.33, *P* < 0.001).

### Personality, health and SWB

#### Social health

Extraversion (*t* = −2.21, *P* < 0.05) and conscientiousness (*t* = −3.52, *P* < 0.001) reduced the negative impact of loneliness on SWB (**Table 3**). The SWB benefits of social network satisfaction were diminished by neuroticism (*t* = −2.07, *P* < 0.05) and increased by greater openness to experience (*t* = 2.23, *P* < 0.05). Neuroticism weakened the positive effect of social network size on subjective well-being (*t* = −2.32, *P* < 0.001).

**Table 3:** Moderating effects of personality on SWB predictors in the top three SWB predictor categories*

		Moderating Effects of Personality (t-scores)				
**Category & Predictor Names**	**+/− with SWB**	**Neuro-ticism**	**Extra-version**	**Conscien-ciousness**	**Agreeabl-eness**	**Openness**
Social health						
*Loneliness*	-	ns	−2.21†	−3.52‡	ns	ns
*Activity satisfaction*	+	ns	ns	ns	ns	ns
*Network satisfaction*	+	−2.07†	ns	ns	ns	2.23†
*Contact frequency*	+	ns	ns	ns	ns	ns
*Network proximity*	+	ns	ns	ns	ns	ns
*Network size*	+	−2.32†	ns	ns	ns	ns
Physical health						
*Overall health*	+	5.98‡	2.90§	ns	ns	2.72§
*Activity limitations*	-	−5.60‡	3.40‡	3.08§	−2.27†	−3.54‡
*Chronic diseases*	-	−4.57‡	ns	ns	−2.61†	−3.35‡
*Chronic illness (y/n)*	-	−5.42‡	ns	2.80§	ns	ns
*BMI*	-	ns	ns	ns	ns	ns
Financial health						
*Making ends meet*	+	4.12‡	ns	ns	ns	−2.54†
*Income*	+	ns	ns	ns	ns	−4.06‡
*Employment*	+	ns	ns	ns	ns	−3.13§
*Cannot afford care*	-	−2.43†	ns	ns	ns	ns
*Cannot afford heat*	-	ns	−2.13†	ns	ns	ns
*Can’t afford food*	-	ns	ns	ns	ns	ns

*Each *P*-value reflects the False Discovery Rate (FDR) adjustment for multiple comparisons.

†*P* < 0.05.

‡*P* < 0.001.

§*P* < 0.01.

#### Physical health

The positive impact of overall physical health on SWB was stronger for participants with more neuroticism (*t* = 5.98, *P* < 0.001), and weaker for those who were more extraverted (*t* = −2.90, *P* < 0.001) and open to experience (*t* = −2.72, *P* < 0.001). Having a chronic illness had a less detrimental effect on SWB for participants with more neuroticism (*t* = −5.42, *P* < 0.001), and a more negative impact on more conscientious participants (*t* = −2.80, *P* < 0.01). Neuroticism also mitigated the adverse impact of chronic illnesses on SWB (*t* = −4.57, *P* < 0.001). However, the negative effects of illnesses on SWB were greater for participants who were more open (*t* = 2.61, *P* < 0.05) and agreeable (*t* = 3.35, *P* < 0.001). Limited physical activity detracted less from SWB for more neurotic participants (*t* = −5.60, *P* < 0.001), and more among those who were more extraverted (*t* = 3.40, *P* < 0.001), conscientious (*t* = 3.08, *P* < 0.01), agreeable (*t* = 2.27, *P* < 0.05), and open to experience (*t* = 3.54, *P* < 0.001).

#### Financial health

The SWB benefits of being able to make financial ends meet was enhanced by greater neuroticism (*t* = 4.12, *P* < 0.001), but reduced by greater openness to experience (*t* = −2.54, *P* < 0.05). Openness also lessened the positive impact on SWB of more employment (*t* = −4.06, *P* < 0.001) and income (*t* = −3.13, *P* < 0.01). Neuroticism lessened the negative impact on SWB of no health care (*t* = −2.43, *P* < 0.05), while more extraverted participants were less affected by being unable to afford heat (*t* = −2.13, *P* < 0.05).

## DISCUSSION

These results demonstrate that personality and health can have significant direct and indirect effects on each other, and on subjective well-being among older adults, and that these effects vary systematically across countries, categories, and individual health factors.

### SWB across countries

Across Europe and Israel, eight tiers of countries differed significantly in subjective well-being, as did Northern and Southern Europe. More broadly, well-being (as measured by the WHI) also differed significantly across six tiers of world regions. These findings are consistent with previous studies [34,49,58], and they indicate that any salutary effects of Southern Europe’s and Sub-Saharan’s warmer weather were eclipsed by other factors, which may include Northern countries’ higher levels of basic services, economic prosperity, civic engagement and social cohesion [59–61].

These differences are unlikely due to sample sizes, as neither SWB nor WHI scores or tier rankings correlated with the number of participants or countries. However, SWB and WHI tiers were associated with life expectancies across European countries and world regions. In fact, these tiers were stronger predictors of life expectancies than their more numerous respective countries and regions. This supports the practical importance of SWB and WHI indices, and the use of tiers to assess the predictive validity of these and other measures.

### SWB across categories

The top five categories with two or more factors (social, physical, and financial health, personality, and demographics) were significantly stronger predictors of SWB than the bottom three (living environment, childhood experiences, and health care). This may reflect the fact that the top five categories, like the SWB measure, were both current and personal, while the bottom three were historical and/or external conditions. These temporal and conditional effects can be assessed by future ratings of SWB predictors along these two dimensions, and by including these ratings as covariates in subsequent analyses.

Social factors were the strongest determinants of subjective well-being, followed by physical and financial predictors. Beyond humans’ social nature, the relative primacy of social health may have reflected participants’ ages and/or the countries from which they were sampled. With an average age of 66 years, over 89% of participants were either retired, unemployed, or permanently disabled, and all were 50+ years old. Thus, the majority were no longer experiencing regular social interactions associated with education, employment, or raising children, which is consistent with their small average social network size of fewer than three people. This may in turn have increased the potential for loneliness, and the relative importance of social interactions for subjective well-being.

The countries in this research are all considered welfare states that provide citizens with support for childcare, education, training, health care, housing, and income [62]. Thus, these participants may have fewer concerns about their physical and financial health than those in other countries. For example, 13.8% of participants in this study reported being unable to afford health care, and 5.8% reported being food insecure. In contrast, about 40% of US adults 50 and older were unable to afford health care in 2015 [63], and about 14% reported being food insecure in 2014 [64]. These potential explanations for the relative impact of social, physical and financial health on subjective well-being can be tested more directly in future research comparing SWB across age groups and socioeconomic structures.

### SWB predictors

The top three social SWB predictors (loneliness, social activity satisfaction, social network satisfaction) were more qualitative indices, while the bottom three (social contact frequency, social network distance, and social network size) were more quantitative measures. This is consistent with a large literature showing significant, positive relationships between social factors and SWB [61,65,66], and a smaller but longstanding group of studies showing stronger SWB effects of qualitative than quantitative social factors [67–69].

The most qualitative physical health factor (overall physical health) was also a stronger SWB predictor than the other, more quantitative physical health indices (*e.g*. number of chronic illnesses). Overall physical health is also a broader construct, the impact of which can be tested more directly by examining the effects of general and specific health evaluations on subjective well-being. BMI was negatively associated with SWB, but this relationship was only marginally significant, suggesting that the impact of BMI on subjective well-being may be more pronounced at higher BMIs. This could be assessed by comparing quartile or tertile splits, and/or by analysing SWB slopes across BMI levels.

Financial health had a positive influence on participants’ subjective well-being. However, the strongest financial SWB predictor was not income, but the ability to make ends meet, suggesting that the SWB benefit of material wealth is based more on sufficiency than maximisation. Employment had less impact than income on SWB, which is consistent with the fact that employment is more distal (and varied) than the income it produces. Inability to afford health care detracted from SWB more than a lack of heat, which was more impactful than periods of hunger. These results may reflect a demand hierarchy for these amenities, which may influence their relative salience.

Neuroticism was the strongest personality predictor of SWB. This is consistent with previous research, both in terms of the link between neuroticism and SWB [70], and its impact relative to the other ‘Big 5′ traits [71]. The greater impact of neuroticism may be due to its negative valence, as negative psychological experiences often have stronger effects on SWB than positive ones [72]. Neuroticism also reflects how individuals perceive (*i.e*. internalise) experiences, while the other personality traits are more externally directed, which may also reduce their impact on subjective experiences. Future research can test these hypotheses more directly by including both positive and negative dispositional measures that are either internally or externally oriented.

Participants’ subjective well-being in this study decreased significantly with age. This is in contrast to prior research in which SWB has decreased during young adulthood, reached a nadir in middle age, and increased later in life [72,73]. This difference may be due to prior studies’ use of singular SWB dimensions, rather than the current aggregate SWB measure. The current participants were also relatively old, and often exhibited lower SWB due to mental, physical, or other difficulties. This is consistent with Hansen and Blekesaune, who have noted that ‘… significant downturns in SWB in advanced age point to limits to psychological adjustment when health-related and social threats and constraints intensify’ [74].

Prior SWB studies did not control for personality, which can have both direct and indirect effects on subjective well-being. As summarised by Graham and Ruiz: ‘Naturally cheerful or happy respondents seem to navigate the aging process – and the stress associated with [it] – more easily than those who are lower in the well-being distribution’ [73]. In this way, personality may help – or hinder – older adults’ health, and its impact on their subjective well-being. It may also affect their ability to cope with their diminishing mental, physical or social status relative to younger individuals – including, perhaps, their younger selves.

Gender differences in previous SWB studies have been mixed, and a recent meta-analysis of 281 effects sizes found no differences in life-satisfaction ratings between males and females [69]. The higher SWB among females in this research suggests that these older women may be less lonely and more socially satisfied, which is consistent with prior research [75]. These women may also be more positive, frugal, physically active and – given that they tend to live longer – less concerned about their mortality.

### Personality and health

Personality traits were associated with participants’ social, physical, and financial health, although effects varied widely across individual factors. Participants with more neuroticism experienced more negative health outcomes and fewer markers of positive health, while the other traits were generally associated with better health and well-being. These results are largely consistent with prior research, both within and across countries [28–30,76].

One exception to this pattern involved extraversion, which was strongly and positively associated with BMI. This may have been due to greater social activity among more extraverted people, much of which may also include food and beverage consumption. This is consistent with research showing that extroverts were more likely to consume higher-calorie foods in social contexts [77]. Extraverts can also be more impulsive [78], which may lead them to engage in more unplanned eating, particularly under stress [79]. The impact of these behaviours on BMI may be more pronounced among older adults, who also tend to be less physically active than younger individuals [80]. These results also suggest that extraverts may be at heightened risk for obesity, and that weight-related interventions for these people should address social situations, food availability, and psychological stress.

Previous studies of the link between neuroticism and BMI are also mixed. Rosa et al. [81] found higher BMIs among more extraverted girls, and Shim et al. [82] found higher BMIs among more extraverted Korean men. However, Arumäe et al. [83] and Sutin et al. [84] reported negative associations between neuroticism and BMI among Estonian and American adults, respectively. Similar future research can clarify these relationships by matching study cohorts for age, gender, nationality, and other characteristics.

Openness to experience was negatively associated with income, and positively related to food insecurity (presumably due to lower income). This contrasts with prior research showing a positive link between openness and income, often attributed to greater creativity, adaptability, innovation or intellectual engagement [85–87]. However, those more open to experience are also often more focused on intrinsic motivations and personal growth than on financial considerations, making them more willing to engage in lower-paying work [88,89]. The latter may be particularly true for older Europeans, whose ages are associated with fewer financial responsibilities than in younger adulthood [90]. These adults also reside in social welfare states [91], giving them greater financial security, and enabling them to pursue more activities that provide relatively little or no compensation.

Greater educational attainment among those more open to experience [92] suggests another potential explanation for their lower income: education-career misalignment. These individuals may choose less career-oriented majors, engage in less strategic career planning, and thus experience more occupational interruptions, flatter career trajectories, and lower incomes. This suggests the importance of examining the moderating effects of age, wealth, and employment histories to better understand the financial impact of openness to experience.

### Personality, health, and SWB

The indirect effects of personality and health on SWB were smaller and less numerous than their main effects, and they were most prevalent among physical health factors. This is not surprising, given the more numerous main effects of social and financial health, leaving less variance to explain in these areas. Neuroticism reduced the positive impact of social network size and satisfaction on subjective well-being. However, it enhanced the SWB benefits of physical health and ability to make ends meet, and reduced the negative impact of chronic illness, activity limitations, and inability to afford health care. These ‘protective’ effects seem unlikely to be attributable to coping strategies, given the historically strong, negative relationship between neuroticism and adaptive coping. Rather, they seem more likely due to neuroticism’s underlying negativity, which may lead people to expect the worst from events and conditions in their lives, leading to ‘pleasant surprise’ in the event of positive outcomes, and a buffering of disappointment in the face of negative ones.

Neuroticism’s negative impact on social health’s ability to enhance SWB may reflect the distal nature of social factors, which may limit the benefits of lowered expectations. For example, because negativity and instability tend to degrade social interactions, the quality of social outcomes – and the resulting SWB – may decline as neuroticism increases. While social isolation – reflected by smaller social networks – is often associated with less well-being [93], more neurotic individuals may view their network sizes as normal, or even desirable [94]. Also, given that social satisfaction is an evaluative index, it may already incorporate expectations and their effects. These potential explanations can be examined in future research by including participant expectations and valuations of health-related outcomes.

Extraversion reduced the adverse effects of loneliness and heat insecurity on SWB, and enhanced the SWB benefits of overall physical health. These are consistent with prior loneliness research results [95,96], and suggest that extraversion may operate at both an emotional and cognitive level, leading people to both feel less lonely, and be less affected by the loneliness they experience. In addition, extraverts’ inclination to seek (and receive) more social support may have been facilitated by better physical health, and it may have dampened the negative psychological impact of heat insecurity (*e.g*. through material support). Conversely, extraversion exacerbated the adverse impact of activity limitations on SWB, suggesting that at least some of these activities were social. To test these explanations, future research could assess the quality and perceived impact of people’s loneliness, the link between their physical health and social support, and their behaviour in response to financial challenges.

Conscientiousness reduced the negative impact of loneliness on SWB, but increased the adverse effects of activity limitations and chronic illness. Conscientious people are more likely to engage in healthy behaviours [97,98], which may in turn buffer SWB from loneliness via distraction and/or better health. In contrast, the higher aspirations, expectations, or need for control associated with conscientiousness may also engender more distress in the face of limitations due to illness or other causes [99,100].

Agreeableness and openness both lessened the negative impact of chronic illness and limited activity on participants’ subjective well-being. This may be due to their associated acceptance, flexibility and social support, all of which can foster better coping and higher SWB under stress [101,102]. Greater openness also enhanced the SWB benefits of social network satisfaction and physical health, presumably for the same reasons [103,104]. Openness to experience also exacerbated the adverse impact of lower employment, income, and ability to make ends meet on their subjective well-being. This indicates that while less financially-oriented career choices have benefits, they can also have material and psychological costs.

### Strengths and limitations

This research included random samples from 18 countries, totalling almost 38 000 participants, increasing its representativeness and statistical power (although the focus on older adults may limit its generalisability to other age groups). It also assessed the independent effects of 17 health factors and five personality measures on a comprehensive measure of subjective well-being, while adjusting for potential Type I error. In addition, using machine learning, GAM, and multiple regression enabled relatively unbiased rankings and effect sizes, as well as direct comparisons between SWB predictors.

By examining the impact of all ‘Big 5′ personality traits on all 17 health factors, this research was able to determine the strength, direction and number of traits associated with each factor. As such, these personality ‘impact profiles’ can help identify individuals who are more (or less) likely to experience these outcomes. This diagnosticity is further enhanced by the moderating effects of personality and health, which can also influence SWB in the absence of significant main effects. Thus, it may also be possible to improve people’s health and subjective well-being by modifying responses associated with these traits.

Although adding happiness to the current SWB measure increased its content validity, neither this nor other aggregate SWB measures can identify potential causes or effects of individual SWB components, nor can they be compared directly with single-component SWB studies. And while the current results are generally consistent with prior research using other SWB measures [105–108], future research including both individual and aggregate indices could address this issue more definitively.

The self-reported and often retrospective nature of the current study variables subject these data to potential bias and the limitations of memory. These likely increased measurement error and decreased statistical power, which may explain some of the null results (although it would also increase confidence in significant findings). A potential source of systematic error is social desirability, or the desire for people to present themselves in a favourable light. As a result, respondents may understate negative attributes (*e.g*. loneliness) and overstate positive ones (*e.g*. subjective well-being), leading to potential confounds. While it is less likely to confound multivariate analyses – and would counter the current unexpected results – it should be controlled for in explanatory research involving self-reports. This can be done easily using the Marlowe-Crowne Social Desirability Scale [109].

This study was also cross-sectional, which limits the ability to make definitive conclusions about the temporal direction of the observed relationships (although this is less of an issue for personality effects). Directionality can be addressed in future longitudinal studies, for which ongoing, multi-wave research like the SHARE project are well-suited. Although the current findings suggest mechanisms for the impact of personality on health – and the effects of both on subjective well-being – the current observational analyses preclude any causal conclusions. However, these mechanisms could be clarified by future research testing more complex moderating and/or mediational models.

Categorical analyses should also be interpreted with caution, for group rankings often depend on which elements are considered part of that group. For example, including loneliness in mental health would have made it the third highest-ranking category, and including hunger periods in physical health rather than financial factors would reverse their respective rankings. This also illustrates the importance of examining these and other factors at the individual level.

Selection bias is another potential limitation of this research. While data were randomly sampled within countries, within-household response rates for Waves 6 & 7 averaged about 90% [41]. Thus, these results may not be entirely representative of their populations. For example, mean incomes in these SHARE data are significantly higher than countries’ national averages, suggesting that less-affluent citizens may be underrepresented.

Finally, as in all research, it is important to consider ethical implications, especially with new and powerful technologies such as AI. For example, training AI algorithms on biased or unrepresentative data can distort research results and conclusions, and lead to inaccurate or unethical assessments, interventions, or outcomes. This potential is heightened by the opacity of many AI systems, which can make it difficult to know how conclusions are drawn or how to detect or correct errors. Artificial Inteligence models may also be used to generate complex patterns without offering explanations or methods for testing them, potentially undermining scientific validity. Thus, it is essential for researchers to explain how machine-learning algorithms are employed, so that their validity and reliability can be assessed [110].

### Policy implications

The current findings suggest that public policies to reduce loneliness among older adults may significantly improve their subjective well-being, and perhaps other aspects of their health. Specifically, programmes that increase social contact and satisfaction may be particularly useful. This might include supporting senior centres and community organisations that enable seniors to share resources and experiences through trips, classes, and other activities [111]. Intergenerational programmes in which older adults mentor students, and visiting/telephone programmes in which (typically younger) volunteers visit or phone socially-isolated seniors may also be helpful [112]. Older adults who are both able and interested may also benefit from time banking, a reciprocal service exchange where people ‘bank’ hours of service, and then draw hours of services they need [113].

Even brief personality profiles may also be useful for programmes and policies by identifying seniors who are more at risk – either directly or indirectly – for various health outcomes. However, the current results also illustrate that these factors also can also have counterintuitive effects. It may also be possible to improve health and well-being by modulating people’s personalities or their associated reactions and behaviours, for while personalities are established early and relatively stable, they are not necessarily immutable.

## CONCLUSIONS

This research has identified many factors and potential mechanisms of influence for the subjective well-being of older adults across countries, categories, and analyses, and it has demonstrated the important role of social, physical, and financial health in this process. Reflecting the complexity of human conditions and experience, these relationships varied widely between personality traits and health factors, illustrating the importance of examining these factors simultaneously. We hope that these findings and future tests of their implications will further enhance our understanding of these effects, and eventually improve the health and well-being of individuals, their societies, and the world.

## Additional material


Online Supplementary Document
