# Supplementary material for: Using Artificial Intelligence to assess the impact of social, physical, and financial health and personality on subjective well-being in a representative, multinational sample of older European and Israeli adults
Source: J Glob Health. 2025 Jun 27;15:04179. doi: 10.7189/jogh.15.04179 (PMC12203629; doi:10.7189/jogh.15.04179)
Supplement: Online Supplementary Document [file jogh-15-04179-s001.pdf]

Supplement to: Moore PJ, Vera Cruz G, Maurice T, Rohrbeck CA, Khazaal K, Goodman FR. Using Artificial Intelligence to assess the impact of social, physical and financial health and personality on subjective well-being in a representative, multinational sample of older European and Israeli adults. J Glob Health. 2025;15:04179.

APPENDIX A: Details related to introduction and method

Section 1: The Subjective Well-Being (SWB) construct

Evaluative SWB. An example of an overall, single-item assessment of evaluative SWB is "Overall, how satisfied are you with life as a whole these days?", which has been used in many social surveys in Europe and North America [1]. The Cantril Self-Anchoring Striving Scale [2] —also used in many worldwide surveys—has respondents indicate their position on a ladder from their "worst possible life" to "best possible life," and is used for both current and projective life-satisfaction assessments [3]. Among the most prominent compound measures of life satisfaction is the Satisfaction with Life Scale (SWLS; [4]), a well-validated Likert scale on which people indicate their agreement with five life statements (e.g., "So far, I have gotten the important things I want in life"). The current SWB measure includes life satisfaction because of its significant contribution to subjective well-being, and because it is among the most ubiquitous SWB components across fields [5]. An additional index of evaluative SWB is the 19-item Quality of Life Scale (CASP-19; [6]), a quality-of-life (QoL) measure designed to assess perceptions of control, autonomy, self-realization and pleasure among older adults, and which is increasingly used in research on aging [7].

Experiential SWB. Perhaps the best-known example of positive experiential SWB is the World Happiness Report (WHR), which includes an annual ranking of happiness in over 140 countries [8, 9]. Similarly, negative emotion—particularly depression—has significant effects on health behaviors and quality of life [10, 11], as well as future physical functioning [12]. The distinction between positive and negative affect is crucial, both because they are independent predictors of life satisfaction and other outcomes [13], and because the value of

experiential SWB research lies not only in enhancing positive experiences, but also in reducing the suffering associated with negative ones.

Section 2: Personality, Health & SWB

Neuroticism tends to be negatively associated with health outcomes, while the other personality traits tend to be positively related to health [14]. In an examination of studies since 2020, health-related outcomes consistently fitting this pattern included cognitive control *(w/ O)* [15], HbA1c levels *(w/ C)* [15], cigarette use *(w/ N)* [16], social activity *(w/ O, E)* [17], fatigue *(w/ O, C, E)* [18], physical activity *(w/ O, C, E)* & anhedonia *(w/ O, E, A)* [19], and health literacy & depression *(w/ O, C, E, N)* [20, 21]. Among the moderating effects of personality on the link between health and SWB, Meuller et al. [22] found that greater conscientiousness reduced the negative impact of disability on subjective well-being. Moderating effects can also strengthen the link between health and SWB, as in Cheuk Long Chan et al.'s [23] finding that people more open to experience benefited more from physical activity in terms of SWB, while higher neuroticism increased the adverse impact of behavioral problems on self-esteem [24].

Section 3: Access to SHARE data

Once collected from participating countries, the data are aggregated and processed for quality control (e.g., identifying & eliminating errors), harmonization (e.g., confirming comparability across countries), and imputation (using the hot-deck method) [25]. After data-collection and quality-control procedures are completed, eligible researcher teams can gain access to the data at the conclusion of the application review process [25]. These research teams are then responsible for generating algorithms for extracting the relevant variables, decoding labels, and transforming the data to address their particular research questions [25].

Section 4: Details on how the data was transformed and analyzed

Data Transformations. While most study variables were kept in their original form, some required transformations and/or aggregation by the SHARE research team. For example, participant education was assessed using the International Classification of Education (ISCED-97 classification), which related educational attainment in each country to an international standard. The original SHARE scales worded in the opposite direction indicated by measures were reverse-coded, and dichotomous variables were recoded as 0/1, with 1 reflecting more of the construct represented by the variables. As confirmed in subsequent analyses, this reverse-coding, while changing the valence, had no effect on the strength of the associations between any of the study variables.

Rank-ordered SWB Predictors. To rank-order the 50 strongest SWB predictors, we used machine learning, which is an application of artificial intelligence whose algorithms build models through iterative analyses of sample data to generate statistical predictions that they are not specifically programmed to test. First, we used the "VSURF" program within the statistical package R [26], whose iterative regression modeling was used to identify the independent, multivariate contribution of each variable to the overall explained variance (R^2) for the model predicting SWB. After this selection process was completed, a machine-learning regression model was built using the Random Forest algorithm [27].

The Random Forest algorithm (which was also used to perform the missing-data imputations) uses a random subset of predictors to test the strength of each predictor in a model through a process called *recursive partitioning*. This process involves first developing a decision tree from the strongest available predictors, and then testing the tree's overall predictive power on a subset of data not used to construct the tree itself (also called "out of bag" sampling). The Random Forest algorithm does this repeatedly, bootstrapping up to thousands of decision trees, and then averaging their results.

In the current study, each Random Forest model was constructed from 500 regression trees, with the number of predictors available for splitting at each tree node equal to one-third the number of predictors. Among other outputs, this process yielded the percent increase in Mean Squared Error (MSE) for each factor in the model predicting SWB, which is the percentage increase in the MSE caused by removing that factor from the model. As such, this measure reflects the extent to which each factor reduces the difference between the predicted and actual SWB values, with higher values indicating stronger, more accurate predictors of SWB, which were then used to rank-order the top 50 SWB predictors. Twenty-five of these predictors (17 health factors, 5 personality factors, and 3 demographic factors) were included in the current study.

In machine learning, the original dataset is split into at least two sets: one to train the model (usually 70–80% of the sample), and the other to estimate its predictive performance (usually 20%-30% of the sample). In the current study, the training set constituted 70% of the sample, while the testing set comprised 30%. The final model predicted a majority of the variance in SWB (R^2 = 58.71%), with very low residual error (RMSE = .41). However, because these MSE analyses do not include main effects or inferential results, additional analyses were conducted to determine the effects sizes and significance levels for these individual predictors of subjective well-being.

Effect Sizes & Significance. To determine the effect sizes and significance of the SWB predictors in this research, we applied Generalized Additive Modeling (GAM) to the continuous and ordinal data [28, 29], while nominal predictors were subjected to Analysis of Variance (ANOVA). A GAM algorithm was chosen because GAMs are better able to fit nonlinear data. Moreover, compared with Generalized Linear Models (GLMs) such as linear regression, GAMs do not assume that the predictive relationship is a simple weighted sum, but rather that it can be modeled by a sum of arbitrary functions of each feature [29]. In

GAM, the beta coefficient from linear regression is replaced with a flexible function that enables the assessment of non-linear relationships. This flexible function—called a *spline*—is a piecewise polynomial that fits multiple, low-degree polynomials to small subsets of values. A primary advantage of splines relative to high-degree polynomials is that they reduce statistical error by reducing the variability between interpolation points [30]. Relative to GLMs, GAMs are also better able to uncover patterns in the data.

The general equation for GAM can be expressed as follows:

$$F(x) = yi = \alpha + f_1 (xi_1) + f_2 (xi_2) + \ldots . f_p(xi_p) + \epsilon i$$

where $f_1, f_2, f_3, \ldots . f_p$ are different non-linear functions on variables X_p. In essence, GAM is a broader, more comprehensive modeling approach that can incorporate non-linear functions—using splines, step functions, etc.—while retaining the ability to test simpler, additive models as well [29]. In this research, multiple GAM models were generated using different fitting parameters, including *families* (i.e., groups of data-modifying functions), *knots* (i.e., the number of spline nodes), *fitting methods* (i.e., model component estimation algorithms), and *optimizers* (curve-smoothing selection algorithms). The final model—which resulted in the best-fit restricted maximum likelihood (REML)—explained over 55% of the variance in participants' subjective well-being.

Section 5: The World Happiness Index (WHI)

The World Happiness Report [8, 9] uses data from the Gallup World Poll and ranks countries based on how people evaluate their lives. The World Happiness Index (or Happiness Score) is based on a Cantril ladder survey question, where respondents rate their life on a scale from 0 (worst possible) to 10 (best possible). The index is not a direct mathematical formula, but rather a regression analysis that correlates life evaluations with several key factors. Here are the main elements used to calculate the index, along with how they're used:

1. GDP per capita

    - Reflects the standard of living.
    - Log of GDP per capita is used to account for diminishing returns of income on happiness.
2. Social support
    - Based on the question: "If you were in trouble, do you have relatives or friends you can count on to help you whenever you need them?"
3. Healthy life expectancy
    - Life expectancy at birth, adjusted for health (e.g., years of healthy life, not just total life span).
4. Freedom to make life choices
    - Based on answers to: "Are you satisfied or dissatisfied with your freedom to choose what you do with your life?"
5. Generosity
    - Measured by responses to: "Have you donated money to a charity in the past month?"
6. Perceptions of corruption
    - Combines responses about corruption in government and business.

- Researchers perform a regression analysis to estimate how much each of these six factors contributes to explaining the variation in life evaluations (on the 0–10 Cantril ladder).
- Each factor is given a coefficient (based on data from 150+ countries) that represents its average impact.
- The final happiness score is a combination of:
    - Actual average life evaluation scores per country
    - Estimated contributions from each of the 6 factors
    - A residual (or unexplained component), which captures other influences not measured by the six variables (e.g., culture, personal values).

Example Breakdown (as seen in the report)

In the report, you might see a country's happiness score broken down like this (sample numbers):

Factor	Contribution
GDP per capita	1.20
Social support	1.50

Factor	Contribution
Healthy life expectancy	0.90
Freedom	0.60
Generosity	0.20
Corruption perception	0.10
Dystopia + Residual	2.00
Total Score	6.50

"Dystopia" is a hypothetical country with the world's worst scores for each factor, added to ensure all countries have a baseline from which to improve.

References

1. Organization for European Cooperation and Development (OECD). OECD Guidelines on measuring subjective well-being. Paris: OECD; 2013. DOI: 10 .1787/9789264191655-en.
2. Glatzer W, Gulyas J. Cantril Self-Anchoring Striving Scale. In: Michalos, A.C. (eds) Encyclopedia of quality of life and well-being research. Springer, Dordrecht; 2014. DOI: 10.1007/978-94-007-0753-5_259
3. Diener E, Kahneman D, Tov W, Arora R, Harter J. Income's differential influence on judgments of life versus affective well-being. Diener E, editor. Oxford, UK: Springer; 2009. pp. 233–246.
4. Diener ER, Emmos R, Larsen R, Griffin S. The satisfaction with life scale. J Pers Assess. 1985;49(1):71–75. DOI: 10.1207/s15327752jpa4901_13
5. Eid M, Larsen RJ. (Eds.). The science of subjective well-Being. New York, NY: The Guilford Press; 2008.
6. Hyde M, Wiggins RD, Higgs P, Blane DB. A measure of quality of life in early old age: The theory, development, and properties of a needs satisfaction model (CASP-19). Aging Ment Health. 2003;7(3):86–94. DOI: 10.1080/1360786031000101157

7. Stoner CR, Orrell M, Spector A. (2019). The psychometric properties of the control, autonomy, self-realisation and pleasure scale (CASP-19) for older adults with dementia. Aging Ment Health.2019;23(5):643-649. DOI: 10.1080/13607863.2018.1428940
8. Helliwell JF, Layard R, Sachs JD, De Neve J-E, Aknin LB, Wang S. (Eds.). World Happiness Report 2022. WHR, 2022. Available from: https://worldhappiness.report/ed/2022/. Accessed: 10 June 2022.
9. Helliwell JF, Layard R, Sachs JD, Neve J-E, Aknin LB, Wang S. World Happiness Report 2023. WHR, 2023. Available from: https://worldhappiness.report/ed/2023. Accessed: 26 Mars 2023.
10. Miniotti M, Lazzarin G, Ortoncelli M, Mastorino L, Ribero S, Leombruni P. Impact on health-related quality of life and symptoms of anxiety and depression after 32 weeks of Dupilumab treatment for moderate-to-severe atopic dermatitis. Dermatol Ther.2022;35(5): e15407. DOI: 10.1111/dth.15407
11. Strine TW, Mokdad AH., Balluz LS, Berry JT, Gonzalez O. Impact of depression and anxiety on quality of life, health behaviors, and asthma control among adults in the United States with asthma, 2006. J Asthma.2008;45(2):123-133. DOI: 10.1080/02770900701840238
12. Shen BJ, Fan Y, Lim KSC, Tay HY. (2019). Depression, anxiety, perceived stress, and their changes predict greater decline in physical health functioning over 12 months among patients with coronary heart disease. Int J Behav Med.2019;26(4):352-361. DOI: 10.1007/s12529-019-09794-3
13. OpenAI, Inc (2024). *ChatGPT-4* [AI language model]. Obtained from https://chat.openai.com.

14. Da Mota MSS, Ulguim HB, Jansen K, Cardoso TA, Souza LDM. (2024). Are big five personality traits associated to suicidal behaviour in adolescents? A systematic review and meta-analysis. J Affect Disord. 2024;347:115–123. DOI: 10.1016/j.jad.2023.11.002
15. Stephan Y, Sutin AR, Luchetti M, Canada B, Terracciano A. Personality and HbA1c: Findings from six samples. Psychoneuroendocrinology. 2020;120. DOI: 10.1016/j.psyneuen.2020.104782
16. Galindo-Donaire JR, Hernández-Molina G, Fresán Orellana A, Contreras-Yáñez I, Guaracha-Basáñez G, Briseño-González O, et al. The role of personality traits on self-medicated cannabis in rheumatoid arthritis patients: A multivariable analysis. Plos One. 2023;18(1):e0280219. DOI: 10.1371/journal.pone.0280219
17. Olaru G, van Scheppingen MA, Stieger M, Kowatsch T, Flückiger C, Allemand M. The effects of a personality intervention on satisfaction in 10 domains of life: Evidence for increases and correlated change with personality traits. J Pers Soc Psychol. 2023;125(4):902–924. DOI: 10.1037/pspp0000474
18. Chan T, Wanigatunga AA, Terracciano A, Carlson MC, Bandeen-Roche K, Costa Jr PT, Simonsick EM, Schrack JA. (2021). Traits and treadmills: Association between personality and perceived fatigability in well-functioning community-dwelling older adults. Psychol Aging. 2021;36(6):710–717. DOI: 10.1037/pag0000631
19. Geerling R, Anglim J, Kothe EJ, Schram MT, Holmes-Truscott E, Speight J. (2023). Relationships between personality, emotional well-being, self-efficacy and weight management among adults with type 2 diabetes: Results from a cross-sectional survey. PLOS ONE. 2023;18(10), e0292553. DOI: 10.1371/journal.pone.0292553

20. Iwasa H, Yoshida Y. Personality and health literacy among community-dwelling older adults living in Japan. Psychogeriatrics. 2020;20(6):824–832. DOI: 10.1111/psyg.12600.
21. Mavrandrea P, Giovazolias T. (2022). The effect of personality on depressive symptoms: The mediating effect of adult attachment. Curr Psychol. 2020; 41(10):6978–6986. DOI: 10.1007/s12144-020-01207-z
22. Meuller S, Wagner J, Wagner GG, Nilam R, Gerstorf D. (2019). How far reaches the power of personality? Personality predictors of terminal decline in well-being. J Pers Soc Psychol. 2019;116: 634-650. DOI: 10.1037/pspp0000184.
23. Cheuk Long Chan B, Luciano M, Lee B. Interaction of physical activity and personality in the subjective_wellbeing of older adults in Hong Kong and the United Kingdom. Behav Sci. 2018;8. DOI: 10.3390/bs8080071
24. Waltz JT, Chou CP. (2023). The components of mental health in young adults: The mediation and moderation effects of self-esteem and personality traits. Curr Psychol. 2023;42:12768-12776. DOI: 10.1007/s12144-021-02513-w
25. Survey of Health Ageing and Retirement in Europe (SHARE, 2024). SHARE Corona Survey Release 9.0.0. Available from: https://share-eric.eu/fileadmin/user_upload/Release_Guides/SHARE_Corona_Survey_Release_Guide.pdf. Accessed: 26 Mars 2025.
26. Genuer R, Poggi JM, Tuleau-Malot C. VSURF: An R package for variable selection using random forests. The R Journal, R Foundation for Statistical Computing. 2015; 7(2): 19-33. Available from: https://hal.archives-ouvertes.fr/hal-01251924/document. Accessed: 10 June 2022.
27. Breiman L. Random forests. Mach Learn. 2001;45: 5-32. DOI: 10.1023/A:1010933404324

28. Hastie T, Tibshirani R. Generalized Additive Models. London: Chapman and Hall, 1990.

29. Wood SN. Generalized Additive Models: An Introduction with R (2nd edition). Chapman and Hall/CRC Press; 2017

30. Hall CA, Meyer WW. Optimal error bounds for cubic spline interpolation. J Approx Theory. 1976;16(2):105-122. DOI: 10.1016/0021-9045(76)90040-X

APPENDIX B: Tables 1-4

Table S1. Descriptive and inferential analysis for SWB across countries

Country*	N (%)	M	SD	Overall Results
Denmark[a]	1783 (4.7%)	.580	.805	*All Countries* $F(17, 43576) = 229.804$ η^2_p=.093 $p < .001$
Switzerland[a]	1531 (4.0%)	.509	.767	
Sweden[b]	1958 (5.2%)	.387	.781	
Austria[b]	2194 (5.8%)	.377	.846	
Luxembourg[c]	1072 (2.8%)	.222	.906	*North* (Denmark, Sweden, Luxembourg, Germany, Belgium) M = .286[x]
Germany[c]	2756 (7.3%)	.217	.879	
Slovenia[d]	3383 (8.9%)	.067	.933	
Belgium[d]	2982 (7.8%)	.026	.929	*East* (Austria, Slovenia, Czech Republic, Poland, Estonia) M = -.023[x,y]
Spain[d,e]	2952 (7.8%)	-.035	.948	
Israel[e]	1575 (4.1%)	-.081	.853	
Czech Republic[e]	2933 (7.7%)	-.098	.851	
France[e]	1875 (4.9%)	-.140	.904	*South* (Spain, Italy, Croatia, Portugal, Greece) M = -.319[y]
Italy[f]	2594 (6.8%)	-.245	1.00	
Croatia[f]	1967 (5.2%)	-.246	1.12	
Poland[f,g]	340 (0.9%)	-.263	1.02	
Estonia[g]	4579 (12.1%)	-.366	.974	
Portugal[g]	482 (1.3%)	-.424	1.00	
Greece[h]	1035 (2.7%)	-.648	1.05	

N = Sample size, M = Standardized SWB index mean, SD = Standard deviation
*Countries and regions without a common superscript have significantly different levels of SWB (p<.05)

Table S2. Descriptive and inferential analyses for WHR well-being across world regions

Region*	N (%)	M	SD	Overall Results
Oceania[a] (Aus/NZ)	2 (1.3%)	7.285	.001	*All Regions*
North America[a,b]	4 (2.6%)	7.239	.132	$F(14, 140) = 22.19$
North Europe[b]	15 (9.7%)	7.137	.370	$\eta^2_p = .689$
South America[c]	12 (7.7%)	6.333	.391	$p < .001$
North Asia (Russia)	1 (0.6%)	5.716	n/a	
East Asia[d]	6 (3.9%)	5.626	.554	
Central America[d]	8 (5.2%)	5.595	.789	
Middle East[d]	18 (11.6%)	5.547	.998	
Southeast Asia[d]	8 (5.2%)	5.443	.931	
South Europe[d]	16 (10.3%)	5.428	.528	
East Europe[d]	8 (5.2%)	5.347	.623	
Central Asia[d]	7 (4.5%)	5.180	.817	
North Africa[e]	7 (4.5%)	4.898	.591	
South Asia[e,f]	7 (4.5%)	4.685	.395	
Sub-Saharan Africa[f]	36 (23.2%)	4.260	.551	

N = Sample size, M = WHR well-being score, SD = Standard deviation
*Regions without a common superscript have significantly different WHR well-being scores ($p<.05$)

Table S3. MSE rankings for top 50 SWB predictor categories with at least two predictors

SWB predictor Categories	Cat	N	N(%)	Mean Rank
Social Health[a]	8	6	100	9.17
Physical Health[a]	9	5	71	14.80
Financial Health[a]	7	6	67	18.33
Personality[a]	14	5	100	22.00
Demographics[a]	1	3	60	22.00
Health Behaviors[a,b]	13	4	36	30.00
Living Environment[b]	5	4	57	37.00
Childhood Experiences[b]	4	7	78	37.86
Healthcare[b]	12	5	45	39.00

Cat = Category N = Sample size, N(%) = Percentage of category predictors

*Categories without a common superscript have significantly different mean MSE rankings ($p<.05$)

Table S4. Descriptive results for predictors in top 3 SWB predictor categories (social factors, physical health, financial status), demographics, and personality characteristics.

SWB predictors	Rank	Cat	N	Scale	M/%	SD(s)
Loneliness	1	8	37991	0-6	0.88	1.38
Social activity satisfaction	2	8	37991	0–10	8.00	1.91
Self-rated general health	3	9	37991	0–5	2.84	1.07
Making ends meet	4	7	37991	1–4	2.89	0.85
Neuroticism	6	14	37991	1–5	2.62	0.98
Social network satisfaction	7	8	37991	0-10	8.96	1.25
Income	8	7	37991	0-2527K	99K	192K
Limited activity due to health	9	9	37991	1–3	46%>1	0.74
Number of chronic diseases	10	9	37991	0–13	1.75	1.60
Social contact frequency	12	8	37991	0–7	4.81	2.17
Extraversion	13	14	37991	1–5	3.51	0.92
Can't afford healthcare	14	7	37991	Y/N	13.8%Y	n/a
Age	15	1	37991	50–102	66.14	9.72
Social network distance	16	8	19395	0–8	3.32	1.51
Social network size	17	8	19395	0–7	2.69	1.48
Employment status	18	6	37991	1-5	27.9%E	n/a
Can't afford heat	20	7	37991	Y/N	6.9%Y	n/a
Have a chronic illness	21	9	37991	Y/N	52.9%Y	n/a
Sex	22	1	37991	F/M	56.4%F	n/a
Conscientiousness	24	14	37991	1–5	4.11	0.78
Education	29	1	37991	0–6	2.93	1.48
Agreeableness	33	14	37991	1–5	3.70	0.79
Openness to experience	34	14	37991	1–5	3.35	0.93
Body mass index	41	9	37991	13-75	27.11	4.62
Can't afford food	46	7	37991	Y/N	5.3%Y	n/a

N = Sample size, M = Composite SWB index mean, SD = Standard deviation,
E = Employed, n/a = not applicable
